# A novel one-class classification approach to accurately predict disease-gene association in acute myeloid leukemia cancer

**DOI:** 10.1371/journal.pone.0226115

**Published:** 2019-12-11

**Authors:** Akram Vasighizaker, Alok Sharma, Abdollah Dehzangi

**Affiliations:** 1 Electrical & Computer Engineering Department, Tarbiat Modares University, Tehran, Iran; 2 Institute for Integrated and Intelligent Systems, Griffith University, Brisbane, Queensland, Australia; 3 Department of Medical Science Mathematics, Medical Research Institute, Tokyo Medical and Dental University (TMDU), Tokyo, Japan; 4 Laboratory for Medical Science Mathematics, RIKEN Center for Integrative Medical Sciences, Yokohama, Kanagawa, Japan; 5 School of Engineering and Physics, Faculty of Science Technology and Environment, University of the South Pacific, Suva, Fiji; 6 CREST, JST, Tokyo, Japan; 7 Department of Computer Science, Morgan State University, Baltimore, Maryland, United States of America; Chuo University, JAPAN

## Abstract

Disease causing gene identification is considered as an important step towards drug design and drug discovery. In disease gene identification and classification, the main aim is to identify disease genes while identifying non-disease genes are of less or no significant. Hence, this task can be defined as a one-class classification problem. Existing machine learning methods typically take into consideration known disease genes as positive training set and unknown genes as negative samples to build a binary-class classification model. Here we propose a new One-class Classification Support Vector Machines (OCSVM) method to precisely classify candidate disease genes. Our aim is to build a model that concentrate its focus on detecting known disease-causing gene to increase sensitivity and precision. We investigate the impact of our proposed model using a benchmark consisting of the gene expression dataset for Acute Myeloid Leukemia (AML) cancer. Compared with the traditional methods, our experimental result shows the superiority of our proposed method in terms of precision, recall, and F-measure to detect disease causing genes for AML. OCSVM codes and our extracted AML benchmark are publicly available at: https://github.com/imandehzangi/OCSVM.

## 1. Introduction

In medicine and pharmacology, it is crucial to understand the mechanism of a disease in order to find an effective treatment method. When dealing with the inherent disorders, finding the disease genes is the first step. Genetic disorders occur due to dysfunction or disease-causing mutations in a single gene or group of genes. Finding disease-related genes experimentally is a time taking process due to the large number of genes. Hence, further biological findings rely on the computational approaches to accelerate experiments to predict novel disease genes from the huge number of unknown genes. Computational methods also decrease the cost of findings the best treatment approaches for patients. To develop these methods, the large number of genes which have been experimentally confirmed as disorder related genes, could be employed as a useful training resource. In addition, there is a group of genes that is not confirmed as disease causing but has a close connection or functional similarities with such genes [1]. For these genes, demonstrating similar attributes with disease-causing genes can indicate possible similarity in their functioning mechanism. Here, our aim is to show disease genes that share common patterns of gene expression-based features can provide a good basis for automatic prediction of candidate disease genes using computational methods.

There is an observation that genes associated with similar disorders are likely to have similar functionality [2]. It is also shown that functionally related genes which caused phenotypically similar diseases can potentially be used to identify disease causing genes [3]. Taking this finding to account, a wide range of two-class classifiers have been employed to tackle this problem in which Decision Tree (DT) [4], K-Nearest Neighbor (KNN) [5], and Support Vector Machine (SVM) [6] are among the most well-known ones.

To tackle this problem, Zhou et al. proposed a knowledge-based approach called Know-GENE to predict gene-disease associations [7]. To build this model they derived gene-gene mutual information from known gene-disease association data and then combined them with known protein-protein interaction networks using a boosted tree regression method [7]. In a different study, Ata et al., proposed N2VKO as an integrative framework to predict disease genes using binary classification [8]. Moreover, Luo et al. [9] and Han et al. [10] predicted disease-gene associations using the joint features and deep learning classifier. All of these techniques used binary classification method to tackle this problem. To this extent, the confirmed disease genes were considered as a positive set and unknown genes as a negative set. However, all of the unknown genes are not necessarily negative. In fact, unknown genes are composed of both positives and negatives. Therefore, such categorization could introduce noise and inaccuracy, and consequently, negatively impact on the performance.

Other methods tried to use unknown genes as unlabeled set (instead of negative ones), and employed positive-unlabeled (PU) learning techniques to improve their results. Mordelet and Vert [11] and Yang et al. [12] proposed algorithms aimed at computing the weighted similarities between samples in unlabeled set and positive samples. They estimated the likelihood of the samples in unlabeled set to be either positive or negative. Jowkar and Mansoori presented a derived reliable set of negative data in order to form a binary classification problem [13]. Later on, Yousef and Charkari, proposed a fusion method to assign genes to disease class and obtained better results [14].

Other studies incorporated network technique analysis to address this issue. For instance, Singh-Blom et al developed the CATAPULT using positive-unlabeled learning influenced by a version of network propagation technique on an Acute Myeloid Leukemia (AML) gene-phenotype network [15]. In another study, Vasighizaker et al. used a novel strategy to extract reliable negatives from a huge number of unlabeled samples in an integrative framework [16]. They introduced a novel method called C-PUGP, based on clustering approach to build a binary classifier and comparatively outperformed traditional methods.

While recent methods indicated promising output in disease gene prediction, they all suffered from several inherent limitations that confined their performance. The main issue with such studies is not having a specific technique to retrieve validated negative data from unlabeled samples to produce reliable result. Therefore, to overcome this limitation, here we propose a novel machine learning method to accurately predict disease causing genes in AML based upon the concept of one-class classification using gene expression data. One-class classification method does not require negative data in the training set. Hence, it could potentially minimize the training error rate, and as a result, is able to perform as an effective and robust solution, compared to binary-class classification methods with unreliable training set.

In general, the main contribution of this paper is proposing one-class classification method to enhancement prediction performance over binary class classification methods by overcoming the issue of noisy unlabeled data as negative samples. Also, we will demonstrate that it can obtain better sensitivity or in other words, better performance in detecting disease causing genes. Moreover, using gene expression as feature helps to build a biologically meaningful approach to determine differences between disease causing genes and other ones. To the best of our knowledge, our proposed method is the first to design a one-class classifier to identify disease genes.

## 2. Materials and methods

In this section, we describe our proposed method for identifying candidate disease genes in Acute Myeloid Leukemia (AML). We aim to assess the impact of a simple one-class classifier to solve this intrinsically one-class problem considering gene expression profile information. The rest of this section explains dataset, features representation, one-class classifier, the learning phase, and the evaluation method.

### 2.1. Datasets and features

#### 2.1.1. Dataset

There are a wide range of databases that consist gene expression data. The two of the most famous are NCBI GEO Datasets and NCBI GEO Profiles. It is possible to search these two datasets according to a specific condition, for example a disease name, or base upon a gene name/annotation. The data discussed here have been extracted from NCBI’s Gene Expression Omnibus [17].

In general, GEO contains 4348 approved datasets in total. In order to compare the healthy and patient samples in AML, we added “Healthy” keyword in our search and obtain 1153 datasets instead of 2674 dataset related to only “AML”. As we interested in doing research on only human genes, we added this filter and narrowed down the result list to 1091. Another important factor to choose the dataset is to select those that also contain gene expression profile. Selecting those datasets that have expression profiling using microarray reduces the number of available datasets to 55.

As this study aims at comparing and classifying patient cells using abnormal expression changes in AML, the other datasets were deleted from the list. Although other studies explore different issues related to AML (e.g. responding of Leukemia cells to a specific inhibitor), here we use a data set that was extracted from a study that focuses on the comparison of normal monocyte and myeloid Leukemia cells and the identification of abnormally expressed genes in AML [18]. In [18], authors indicate the over-expressed genes (compared with the other genes) as the potential therapeutic targets. Therefore, this dataset exactly matches our main goal and meet the requirement of our experiments to classify and identify genes with the abnormal expression changes.

Moreover, in order to have a more significant p-value, we required a relatively large dataset in terms of the size. Considering each patient as a sample, the more sample size the more reliable result will achieve be obtained. Also, in order to guarantee the reliability and quality of the dataset, we tried to employ a dataset which has been widely used in the literature with proper quality check [18]. Hence, this dataset as one of the most updated ones is considered for our experimentations.

The dataset used in this article made publicly available by Stirewalt et al. [18] and is accessible through GEP accession number GSE9476. The dataset comprises of gene expression levels of 38 normal and 26 acute myeloid leukemia (AML) patients, and was obtained using the Affymetrix Human Genome U133A microarray platform with accession number GPL96.

After obtaining the data, the next step is to form our final benchmark dataset for statistical analysis. To this end, we first set up a measure to select a set of significant genes in the disease upstream process. Depending on the literature, there are different measures to define. A simple method is random selection as it was done in [19]. Another option is based on Log Fold Change and adjusted p-value, where the positive set consists of genes that are more differentially expressed and the remaining genes which are less differentially expressed are used to form the unlabeled set. We consider genes which have log fold change value less than −1 or greater than +1, together with an adjusted p-value less than a threshold of 0.05 as the top differentially expressed genes. As a result, the gene expression matrix for a total of 1174 positive genes and 1300 unlabeled genes are collected from the original dataset.

The top list of positive genes obtained in the dataset preparation process are then employed for the classification method. Therefore, we could then evaluate the similarity between the genes in the unlabeled set and the characteristics of each gene in the positive set. If a gene in the unlabeled set met the similarity measurement in the model, this gene is listed in the candidate disease gene. This dataset is provided as supplementary material (S1, S2, and S3 Files) to this article to make future comparisons feasible and reliable.

#### 2.1.2. Biological feature representation

It has been shown that gene expression levels in disease genes have predictable pattern in different diseases [20]. Hence, we use gene expression profiles in a dataset of AML to characterize genes with their corresponding feature vectors. Each gene *g*_*i*_ is represented as a vector *v*_*i*_ which consists of gene expression levels explaining the process of synthesizing information in the genes into the gene products. The gene expression profile is a collection of gene expression levels which is measured in different conditions or times. These conditions are dependent on different diseases and experiments. For example, the sequence of gene expression levels {*x*_1_, *x*_2_, …, *x*_*m*_} which belongs to gene *X* is defined as its gene expression profile. One of the main feature of gene expression profile is that it can be calculated for different genes. For example, *x*_1_, *y*_1_, …, *z*_1_ are measured simultaneously under the particular experimental environments and conditions. We consider each expression of a gene, i.e. *x*_*m*_, as a feature in the feature vector. In the other word, the gene expression profile *G*_*p*_ = {*x*_1_, *x*_2_, …, *x*_*m*_} is a feature vector. The final dataset is presented as a *N* × *P* matrix format with W = {*w*_*np*_} where *w*_*np*_ denotes the expression values of the gene *n* in the *p-th* sample.

### 2.2. One-class classifier

**Fig 1:**
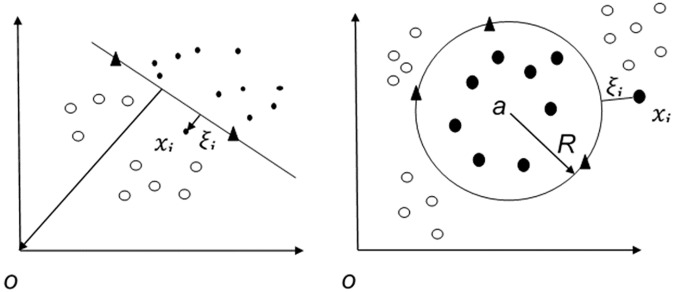
The general architecture of one-class classification of SVM, OCSVM (Left) and SVDD (Right).

Here we introduce one-class support vector machines (OCSVMs) as a means of identifying and predicting the presence of disease genes in AML samples in the unlabeled set. “One-class classification” term was employed first by Moya et.al [21] in 1993. Others, employed outlier detection, novelty detection, and concept learning for this type of learning problem. All of these terms inspired by different application of one-class classifiers. In one-class learning problem, the positive or target, which are either more abundant or clearly defined are labeled correct while the other negative or non-target samples are either non-existence or are very few and classifying them are not of any importance. In the prediction of disease genes, our main aim is to explore and detect disease genes (target class). OCSVM as a semi-supervised algorithm, learns a decision function for classifying new data as similar or different to the training set. The classifier tries to detect a single class and reject the others. The OCSVM method was introduced by Schölkopf et al. [22]. The idea behind the OCSVM is to describe target class by a function that maps the most part of it to a region where the function is nonzero. The problem is solved by finding a separating hyperplane (decision function) with maximum distance from the region containing target class (as shown in Fig 1, Left). The primal form of OCSVM is as follows:

$${\text{min}}_{w,\rho ,\xi }\frac{1}{2}w^{t}w-\rho +\frac{1}{\nu_{l}}\sum_{i=1}^{l}\xi_{i}$$ (1)

Subject to:

$$w^{t}\phi (x_{i})\geq \rho -\xi_{i}$$

$$\xi_{i}\geq 0,i=1,\ldots ,l$$

Where *w* and *ρ* are linear decision function parameters for *l* instances. Also, *ξ* is the cost of training with undergoing a little penalty, and $\sum_{i=1}^{l}\xi_{i}$ is the error rate of training. The penalty parameter or rejection fraction of the classifier, *v* ∈ (0, 1), is employed in order to control the tradeoff between the complexity of the model, $\frac{1}{2}w^{t}w-\rho ,$ and the error rate of the classifier. Also, *ϕ*(*x*_*i*_) is the mapping function.

There are another OCSVM formulation, namely, Support Vector Domain Description (SVDD) which introduced by Tax and Duin [23]. In this model, they find a hypersphere with minimal radius containing only the target class samples and samples lying outside are outliers (as shown in Fig 1, Right). It was shown in [24] that when working with isotropic kernels, for example the Radial Basis Function (RBF), Gaussian kernels, and normalized data, both OCSVM and SVDD method yield the same solution in most cases.

### 2.3. Building classification model

In this study, we use OCSVM with different kernels. Among different kernels, the best model in which yields the lowest classification error is using linear kernel. To this end, we suppose *G*_*p*_ = {*x*_1_, *x*_2_, …, *x*_*m*_} as gene expression profiles of a disease gene. To avoid bias in sampling, we remove outliers. After that, all the feature vectors of instances are scaled according to Min-Max formulation presented by Eq (1)

$$x^{\prime }=\frac{x-x_{min}}{x_{max}-x_{min}}$$ (2)

Where *x*′ ∈ [0, 1], and *x*_*min*_ and *x*_*max*_ are the minimum and the maximum values of the features, respectively. Also, in order to minimize the overfitting of the model, 10-fold cross validation is carried out in all of the experiments. As mentioned earlier, the binary-class classifiers treat unlabeled set as negative. In order to removing the bias effect, we investigate the performance of them using the balanced datasets such that |*P*| = |*N*|, so that we have a balanced dataset following the setup of [4], [6], and [5].

In many applications, it is defined as a requirement to being able to decide whether a new sample belongs to the same distribution as existing samples (inlier), or should be considered as different (outlier). In this case, the training data contains outliers which are defined as instances that are far from (not similar) the others. Inliers are labeled positive, while outliers are labeled negative. The predict method makes use of a threshold on the scoring function computed by the estimator. A positive score for a class indicates that *x* is predicted to be in that class. A negative score indicates otherwise. The decision function is also defined from the scoring function, in such a way that negative values are outliers and non-negative ones are inliers.

### 2.4. Evaluation method

Since the training data do not contain any negative data, trained model can only report accurately true positive rate and it is hard to guarantee high accuracy when model apply to a separate validation set consisting of both positive and unlabeled data. In this section, we describe the performance metrics used in this article and then explore the significant challenge of one-class classifier regarding the metrics.

#### 2.4.1 Performance evaluation metrics and the main challenge of one-class classification

The confusion matrix is normally used as the criterion to assess the performance of the binary classification algorithms. It includes the four elements, true positives rate (TP), the number of positive cases correctly classified; true negatives rate (TN), the number of negative cases correctly classified; false positives rate (FP), the number of misclassified negative cases; and, false negatives rate (FN), the number of misclassified positive cases. Also, measures precision, recall, and F-measure which are defined as Eqs 3–5.

$$precision=\frac{TP}{TP+FP}$$ (3)

$$recall=\frac{TP}{TP+FN}$$ (4)

$$F-measure=\frac{2*precision*recall}{precisio+recall}$$ (5)

One of the main significant challenge in one-class classification context is the evaluation of the classifier [25]. According to the precision formula, the absence of negative samples in training set makes it impossible to estimate such a classical performance measures using this formula. The confusion matrix is required to calculate all four elements. In situations that there are no negative samples, only TP and FN can be calculated. Therefore, according to the definition, only recall can be estimated but calculating precision requires FP which is not available. Alternatively the following formula proposed in [26] is introduced as the model selection criteria:

precision=p=P[Y=1|f(x)=1] (6)

recall=r=P[f(x)=1|Y=1] (7)

Where X and Y present the input vector and the real label vector, respectively. According to these criteria, recall can be estimated as the proportion of correctly predicted positive data from the only positive data in the validation set, and *P*[*f*(*x*) = 1] can be estimated as the proportion of predicted positive data from the whole validation set, consist of positive and unlabeled data [27]. Hence, from a probabilistic point of view, the recall is the probability that a real positive case, *Y* = 1, is correctly predicted as positive by the classification function *f*(*x*), and the precision is the probability of a situation in which a predicted positive instance is really a positive instance, *Y* = 1. We apply this idea to our method and compare the results.

## 3. Results and discussion

Here we present the experimental results achieved using OCSVM. We also compare the results achieved from traditional two-class classifiers with our new one-class classification model. For running all the experiments, we use a PC equipped with 5 Intel cores CPU with 2.4GH frequency and 4G of RAM. The system requirement for running our model demonstrates the efficiency of training OCSVM.

### 3.1. The evaluation of the proposed method

In this part, we aim at investigating how well the proposed method can produce more reliable results compared to the other methods by presenting the achieved results for the disease in question, AML cancer. In order to test our proposed model, we separate positive data to 70%-30% to employ as training and testing sets, respectively. In this way, we make sure that an independent test set has never been used for parameter tuning to avoid overfitting. We also employ unlabeled set to detect positive genes among unlabeled ones.

#### 3.1.1. The results of the OCSVM

**Fig 2:**
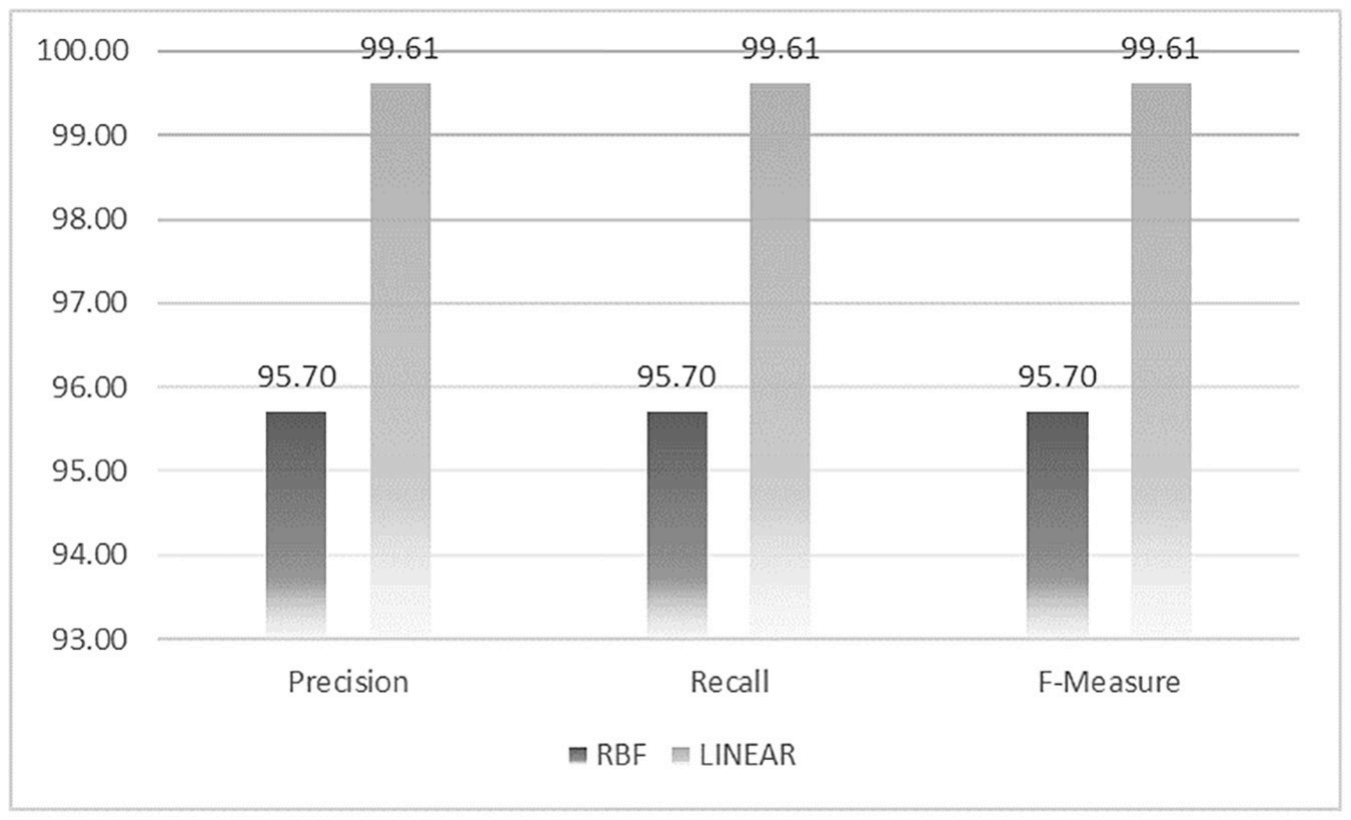
The results of OCSVM Method with linear and RBF kernel.

Here we present the results of our proposed method. As it is shown in Fig 2, using linear kernel, we obtained better results compared to RBF kernel. Therefore, we report the results of the method using linear kernel. The results presented in Table 1 shows the precision, recall, and F-measure using linear and RBF kernel as 99.6% and 95.7%, respectively. As also shown in Table 1, OCSVM is an even-break point support method since precision and recall have almost equal values. It means that this method does not sacrifice precision in favor of recall and conversely.

**Table 1:** The results of OCSVM with linear and RBF kernel.

Kernel	Precision	Recall	F-measure
**RBF**	95.70	95.70	95.70
**Linear**	**99.61**	**99.61**	**99.61**

### 3.2. Comparing the results with previous studies

The most significant aspects which are considered in disease-gene prediction are: 1) the classification method, 2) the biological features, and 3) the feature representation methods. As the all previous studies have utilized binary classifiers, in this study, we employ one-class classifier as the classification method to solve this intrinsic one-class problem and to compare it with the well-known two-class classifiers as well as positive-unlabeled (PU) learning methods. To the best of our knowledge, our proposed method is the first to design a one-class classifier to identify disease genes. As discussed in the Introduction Section, studies that used PU learning techniques also used two-class classifiers for their classification method [11–13, 16]. Among methods in which used PU learning, recently proposed model called C-PUGP is opted for comparison. In the case of two-class classifier, we also choose three state-of-the-art classifiers which have been widely used for this problem (SVM, KNN, and DT). To conduct this comparison, we use feature vectors that is derived from gene expression information which has been shown effective to tackle this problem. Our main aim is to show the preference of one-class classifier compared to the recent (C-PUGP) and most widely used classical classifiers (SVM, KNN, and DT) using same set of features. In future, our aim is to extend this work using a larger benchmark to be able to directly compare our method with other methods such as deep learning techniques.

**Fig 3:**
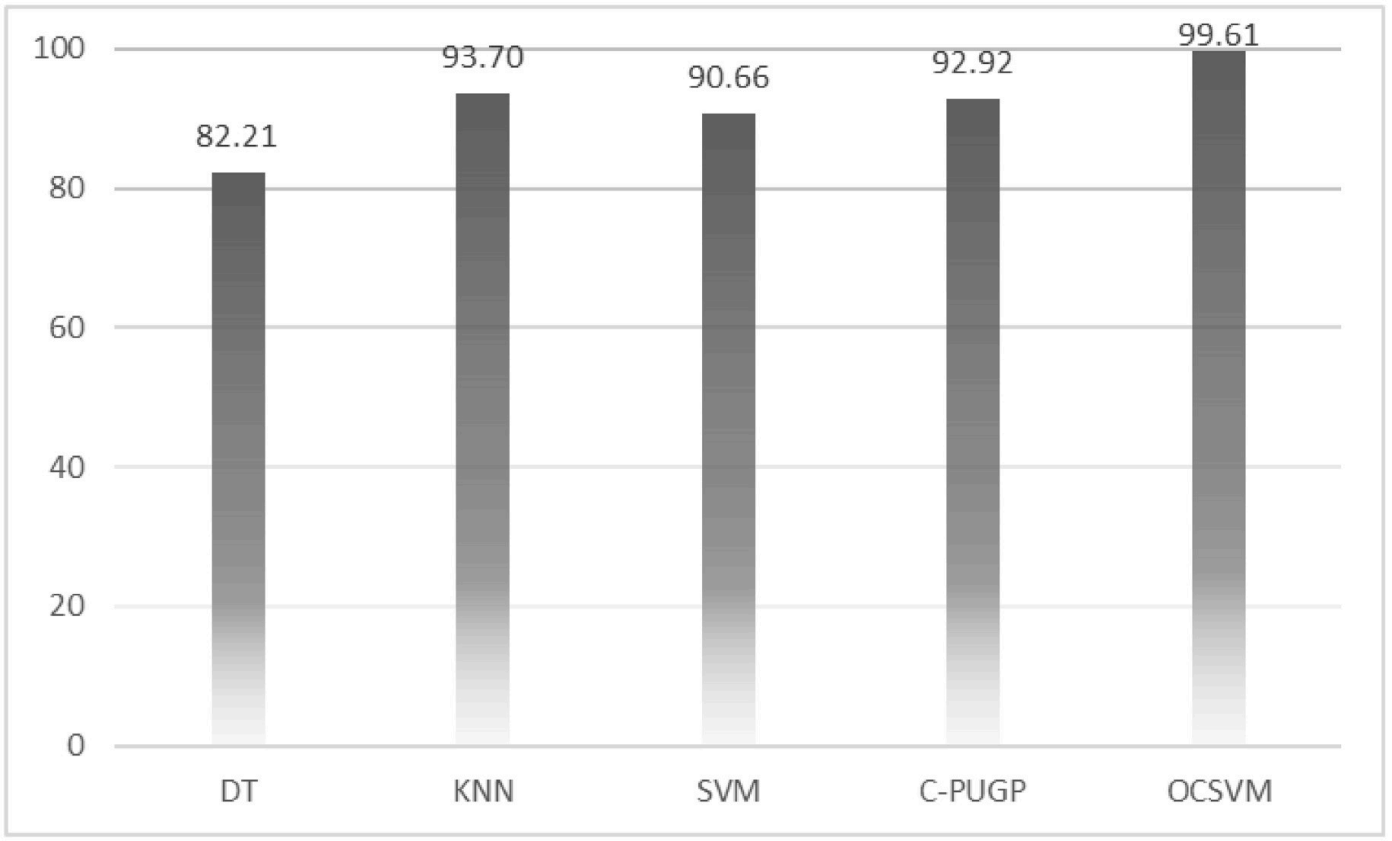
The comparison between OCSVM and other methods for precision.

**Fig 4:**
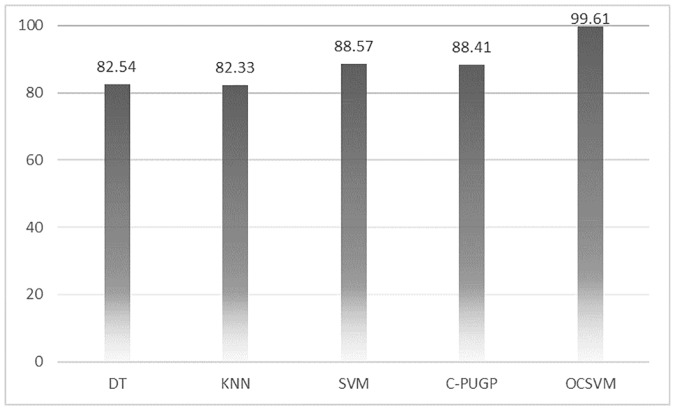
The comparison between OCSVM and other methods for recall.

**Fig 5:**
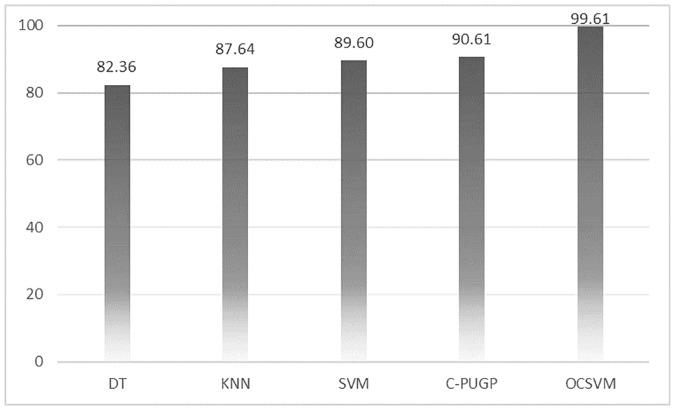
The comparison between OCSVM and other methods for F-Measure.

We show the comparison between our proposed method and three state-of-the-art traditional binary-class classifiers such as those employed in Smalter’s method [6], Xu’s method [5], and PROSPECTR [4], as well as Positive-Unlabeled learning method (C-PUGP) [16]. All the five methods used the same group of training and test set for fair comparison and results are presented in Table 2 and the relevant charts are depicted in Figs 3–5.

**Table 2:** The comparison among methods (precision, recall, and F-measure).

Method	Precision	Recall	F-measure
**PROSPCTOR** [4]	82.21	82.54	82.36
**Xu’s method** [5]	93.70	82.33	87.64
**Smalter method** [6]	90.66	88.57	89.60
**C-PUGP** [16]	92.92	88.41	90.61
**OCSVM**	**99.61**	**99.61**	**99.61**

According to the precision and recall shown in Table 2, our one-class model is able to predict the positive instances with the highest performance. As shown in this table, KNN and C-PUGP achieve 93.7% and 92.9% precision, respectively, approximately 5% and 6% lower compared to those from the OCSVM. Also, reported recall for C-PUGP and SVM are both almost 88% which are and roughly 11% lower compared to those from the OCSVM. The overall results indicate that the C-PUGP is ranked after the OCSVM, according to its F-measure value of 90.6% which is 9% lower than the OCSVM. It also shows that C-PUGP can handle the unlabeled genes for distinguishing the hidden disease genes in the test set better than other methods. Also, it can be seen in Table 2 that the minimum value for precision and recall for other two-class classifiers are reported for DT with 82.2% precision and for KNN with 82.3% recall. Moreover, Table 2 confirms that DT reports the lowest value for F-measure as well (82.3%) which is 17.2% lower than those from the OCSVM. In general, better results achieved using the OCSVM compared to the other methods demonstrates the benefit of one-class classification over the conventional binary-class classification method.

While the binary-class classifiers employ noisy unlabeled set as negative set which is not fully reliable, one-class classifier enjoy the advantages of only using disease genes (one class) without considering other class (non-disease genes) to produce more reliable results. Since there is no proven method to separate negative observations in the unlabeled set, the classifiers that use the unlabeled set in the learning phase are more prone to error. Therefore, it is the advantage of the OCSVM method in which it uses the disease gene information to find disease genes, and unlabeled genes do not appear in the training set to build the model. Indeed, biologists do the same way in their experiments as well. For them, finding non-disease genes is not an aim and priority. Instead they are interested in finding disease genes based on the signs appear on the relevant disease genes. Moreover, using gene expression profile leads to a high performance as it can be seen from the results. In other words, both feature set (biological view) and method (computational view) have significant effect on the performance of identification of candidate disease gene. The OCSVM method and our extracted AML benchmark are publicly available at: https://github.com/imandehzangi/OCSVM.

## 4. Conclusion

Machine learning approaches have been widely used to predict novel disease-causing genes. Despite substantial advancement in disease gene recognition, there are still many genes that are yet to be discovered. Since there are no real negative samples in this problem, selecting a suitable computational method that could encounter this inherent limitation can be considered as a solution with maximum reliability. In this paper, we propose OCSVM as a one-class classification method, to classify and predict novel disease genes from a large number of unknown genes using gene expression profile information. This model is build using one-class model of the support vector machine classifier. As the ultimate goal of one-class classifier is separating positive samples from other ones, we use it to find disease genes (positive set) which has the similar objective. In this specific problem, the main aim is to identify disease genes while identifying non-disease genes are of less or no significant. Here an independent test set is employed to evaluate the proposed method. We also employ unlabeled set to detect positive genes among unlabeled ones to avoid overfitting. The results achieved using our proposed method indicate significant improvement over those methods found in the literature (6.6%, 11.1%, and 9% in terms of precision, recall, and F-measure, respectively).

We believe our model can be used in wide range of problems in Bioinformatics and computational biology. OCSVM codes and our extracted AML benchmark are publicly available at: https://github.com/imandehzangi/OCSVM. Here we conducted our experiments for the AML cancer to investigate the ability of one-class classifier to predict disease-gene association. However, in our future work, we will extend our experiments and investigate the use of the one-class classifier for other diseases.

## Supporting information

S1 File Supplementary material.docx. Detailed Information of our extracted Dataset.
(DOCX)

S2 File Positive_samples.csv. The gene expression matrix for a total of 1174 positive genes.
(CSV)

S3 File EXP.csv. The complete gene expression matrix.
(CSV)
