# Supplementary material for: A novel one-class classification approach to accurately predict disease-gene association in acute myeloid leukemia cancer
Source: PLoS One. 2019 Dec 11;14(12):e0226115. doi: 10.1371/journal.pone.0226115 (PMC6905554; doi:10.1371/journal.pone.0226115)
Supplement: S1 File — Detailed Information of our extracted Dataset. (DOCX) [file pone.0226115.s001.docx]

**Supplementary Material**

We extract EXP.csv from the high throughput microarray downloaded dataset which contains expression level of 22283 genes measured for 64 samples including AML, CD34+, BM, and PB. Table S.1 shows the number of samples in each group.

**Table S.1: Numbers of samples for AML, CD34, BM and PB**

|  | **AML** | **CD34+** | **BM** | **PB** |
| --- | --- | --- | --- | --- |
| Number of samples | 26 | 18 | 10 | 10 |

AML: Acute myeloid leukemia

BM: Bone Marrow

PB: Peripheral Blood

CD34+ : Selected blood cells which are more similar to AML cells compare with the BM and PB cells.

We select significant genes based on adjusted p-value and log fold change value. To this end, at first, we calculate the adjusted p-value and log fold change for all genes in the original dataset. Then, we select those genes with expression level higher than threshold as our final positive gene set. This set consists of 1175 genes which is provided as supplementary material.

Adjusted p-value are chosen based on False Discovery Rate (Type $I$ Error). It means that we try to decrease Type $I$ Error, with threshold of 0.05 for p-value to obtain more significant genes in AML with high confidence.

We use “Limma” Library in R packages to analysis differentially expression based upon log fold change and adjusted p-value.

The following Table S.2 listed some samples in the raw data:

**Table S.2:** **Some Samples in the raw dataset**

| GSM239170 | BM_CD34_R000030 |
| --- | --- |
| GSM239323 | BM_01000 |
| GSM239345 | FHCRCAML72 |
| GSM240430 | PB_DON005 |
| GSM240431 | PB_DON034 |
| GSM240500 | PBSC_CD34_K015367 |
| GSM240502 | PBSC_CD34_K015567 |
